# Novel biomarkers for CKD risk stratification: a literature review

**DOI:** 10.1590/2175-8239-JBN-2025-0109en

**Published:** 2026-01-09

**Authors:** Sariya Khan, Aleena Zobairi, Elaf Rehan, Ashraf Hussein Mohammed

**Affiliations:** 1 Batterjee Medical College, General Medicine Practice Program, Jeddah, Saudi Arabia.; 2 Mansoura University, Mansoura, Egypt.; 3 Saudi German Hospital, Nephrology Department, Jeddah, Saudi Arabia.

**Keywords:** Renal Insufficiency, Chronic, Bioindicator, Biomarkers

## Abstract

**Introduction:**

Chronic kidney disease (CKD) is a progressive illness with high morbidity and mortality that warrants early and accurate risk stratification for optimal management. The traditional biomarkers, serum creatinine and estimated glomerular filtration rate (eGFR), are insufficient for detecting early CKD and long-term prognosis. Novel biomarkers have emerged as effective tools to complement CKD diagnosis, prognosis, and therapeutic monitoring.

**Aim:**

The aim of this research was to determine the potential of novel biomarkers in CKD risk stratification and their clinical significance for improving early detection, monitoring disease progression, and developing individualized treatment strategies.

**Methods:**

A literature review was conducted by searching the PubMed, Scopus, and Embase databases to identify studies on novel CKD biomarkers, including cystatin C, neutrophil gelatinase-associated lipocalin (NGAL), kidney injury molecule-1 (KIM-1), and specific microRNAs.

**Results:**

Emerging evidence suggests that novel biomarkers provide superior predictive abilities compared to traditional markers. Cystatin C is more accurate in kidney function estimation, whereas NGAL and KIM-1 are markers of early kidney injury. MicroRNAs show potential in distinguishing between CKD subtypes and predicting disease progression. Clinical application of these biomarkers may enhance CKD risk stratification, allowing more targeted intervention strategies.

**Conclusion:**

New biomarkers in CKD risk stratification represent a watershed moment in nephrology, offering improved early detection and prognostic accuracy. While promising, additional large-scale research and clinical validation are required before they can be used routinely.

## Introduction

One of the major causes of non-communicable disease morbidity and mortality is chronic kidney disease (CKD). CKD is a long-term condition in which the kidneys’ filtering units (nephrons) gradually lose the ability to excrete waste products and regulate important metabolic processes, leading to a buildup of toxins, fluid retention, and imbalances in electrolytes like potassium and sodium. It is defined by a glomerular filtration rate (eGFR) below 60 mL/min/1.73 m^2^ or the presence of kidney damage (e.g., proteinuria) for more than three months^1^. The leading causes of CKD include diabetes mellitus, hypertension, and glomerulonephritis, with risk factors such as aging, obesity, smoking, and genetic predisposition contributing to its progression. Without timely intervention, CKD can progress to end-stage kidney disease (ESRD), requiring dialysis or kidney transplantation for survival^2^.

CKD is one of the leading causes of death and suffering in the twenty-first century. Its prevalence is increasing globally, and it is now the seventh leading risk factor for mortality worldwide, being expected to reach the 5^th^ position. It is one of the few non-communicable diseases that have shown an increase in associated deaths over the past 2 decades^3,4^. The description of the different stages of CKD is summarized in Table 1. CKD is a degenerative illness that impacts about 800 million people globally, or more than 10% of the global population. An estimated 80% of the global burden occurs in low- or middle-income countries (LMICs), with 25% affecting individuals under 60 years old. CKD represents a substantial burden in these regions, which are often ill-equipped to manage its consequences. In contrast, high-income countries typically allocate from 2 to 3% of their annual healthcare budgets to treat end-stage kidney disease, despite these patients representing under 0.03% of the total population^5^.

**Table 1:** Description CKD stages^3,4^

CKD stage	Characterization	Glomerular filtration rate (eGFR) (mL/min/1.73 m^2^)
Stage 1	Normal kidney function, but some signs of kidney damage, such as protein in urine.	≥ 90
Stage 2	Mildly reduced kidney function	60–89
Stage 3b	Moderate reduction in kidney function	30–44
Stage 3a	Moderate reduction in kidney function	45–59
Stage 4	Severe reduction in kidney function	15–29
Stage 5	Kidney failure	< 15

CKD imposes a significant economic and social burden worldwide, especially in LMICs, due to the high costs of dialysis and transplantation. Many patients in LMICs lack access to affordable care, leading to high mortality rates among CKD patients who cannot afford life-saving treatments^5^.

**Figure 1:**
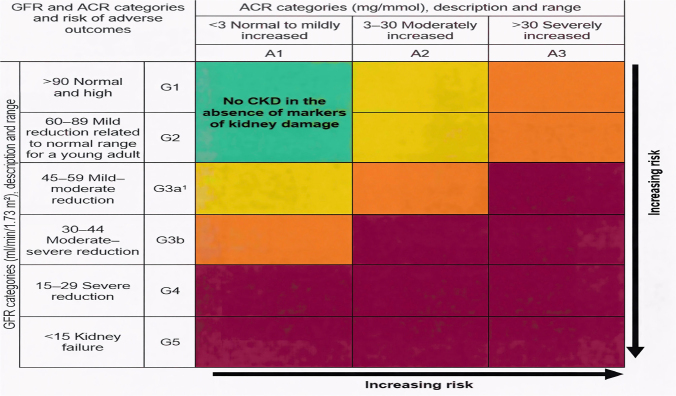
The different stages of CKD and ACR^11^.

Early detection and risk stratification are crucial in CKD management, significantly affecting disease progression and patient outcomes. Identifying CKD in its initial stages allows for timely intervention, which can slow or even halt its progression to more severe stages. It also improves the prognosis by enabling better management of the associated conditions like hypertension and diabetes, which reduces the risk of complications, including cardiovascular disease. By addressing these underlying conditions early, healthcare professionals can help reduce the risk of complications, including cardiovascular disease, stroke, and heart failure conditions that are more common in CKD patients and contribute to increased morbidity and mortality^6,7^. Moreover, the financial aspect of early intervention is significant for treating CKD at its initial stages, as it is not only more effective but also considerably more cost-efficient than managing advanced stages of the disease^7^. The different stages of CKD and ACR are described in Figure 1.

Consider using eGFRcystatinC for people with CKD G3aA1 (see KDIGO recommendations 1.1.14 and 1.1.15).

Abbreviations – ACR: albumin:creatinine ratio; CKD: chronic kidney disease; GFR: glomerular filtration rate. Adapted with permission from Kidney Disease: Improving Global Outcomes (KDIGO) CKD Work Group (2013) KDIGO 2012 clinical practice guideline for the evaluation and management of chronic kidney disease. Kidney International (Suppl. 3): 1–150.

Traditional markers like serum creatinine, eGFR, and proteinuria have been essential in diagnosing and monitoring CKD. However, they have several limitations that can impact the effectiveness in early detection, diagnosis, and management^8,9,10^.

The purpose of this review is to explore and evaluate the potential of novel biomarkers for improving risk stratification in CKD. Emerging biomarkers across various categories, including risk assessment and monitoring of CKD progression, are evaluated. By focusing on the clinical utility and challenges of these novel biomarkers, the review seeks to provide insights into how they could revolutionize CKD management and help optimize personalized treatment strategies. Ongoing clinical trials are focused on validating novel biomarkers for CKD risk assessment, improving early detection, and integrating biomarkers into clinical practice, while future research should involve standardization, long-term studies, and cost-effectiveness evaluation to facilitate widespread adoption.

## Methods

This article is a narrative review that synthesizes new evidence on the early prediction of CKD using new and updated biomarkers. A literature search was conducted in PubMed/MEDLINE, Web of Science, and Google Scholar from the databases’ inception to February 2025. The keywords of primary importance were “CKD biomarkers”, “cystatin C”, “neutrophil gelatinase-associated lipocalin (NGAL)”, “kidney injury molecule-1 (KIM-1)”, and “microRNAs”. Boolean operators (AND/OR) and truncations were applied where relevant. We considered studies published in English and that addressed novel biomarkers in CKD. We considered original research articles (case-control, cohort, cross-sectional, and case series), systematic reviews, and narrative reviews. As this was a narrative review, no formal screening, inclusion, or exclusion process was applied. Articles were selected based on their relevance to the review objectives and the authors’ judgment, drawing from both database searches and previously known literature. No restrictions were placed on study design or year of publication, and the inclusion of sources was determined by their perceived contribution to the thematic synthesis rather than adherence to predefined eligibility criteria.

## Traditional vs Novel Biomarkers in CKD Risk Stratification

The diagnosis and monitoring of CKD have benefited greatly from traditional biomarkers, such as urine albumin, serum creatinine, and eGFR. These biomarkers, however, have a number of drawbacks that may affect their ability to detect the disease early, accurately evaluate it, and predict its prognosis^11,12^.

Serum creatinine is a very common traditional biomarker. Its dependence on muscle mass makes it an unreliable indicator of kidney function, which can cause misclassification in people with unusual high or low muscle mass. Furthermore, it is not appropriate for early CKD identification because its levels only increase once substantial kidney damage has occurred. Its levels are further impacted by non-kidney factors such as diet, medicines, and hydration^13,14^.

Similarly, eGFR, which is based on serum creatinine, has drawbacks such as being sensitive to non-kidney variables and muscle mass. Age and ethnicity differences can also result in misclassification. Furthermore, eGFR is not reliable for people with unusual eating patterns, changed metabolisms, or extreme body sizes. Urinary albumin is also used, but it can be affected by temporary variables like illnesses and physical activity, which can result in false positive results. It does not detect interstitial or tubular injuries and mainly identifies glomerular damage. Furthermore, the presence of albuminuria does not necessarily predict CKD, and some individuals may experience disease progression even without albuminuria^13,14^.

**Figure 2:**
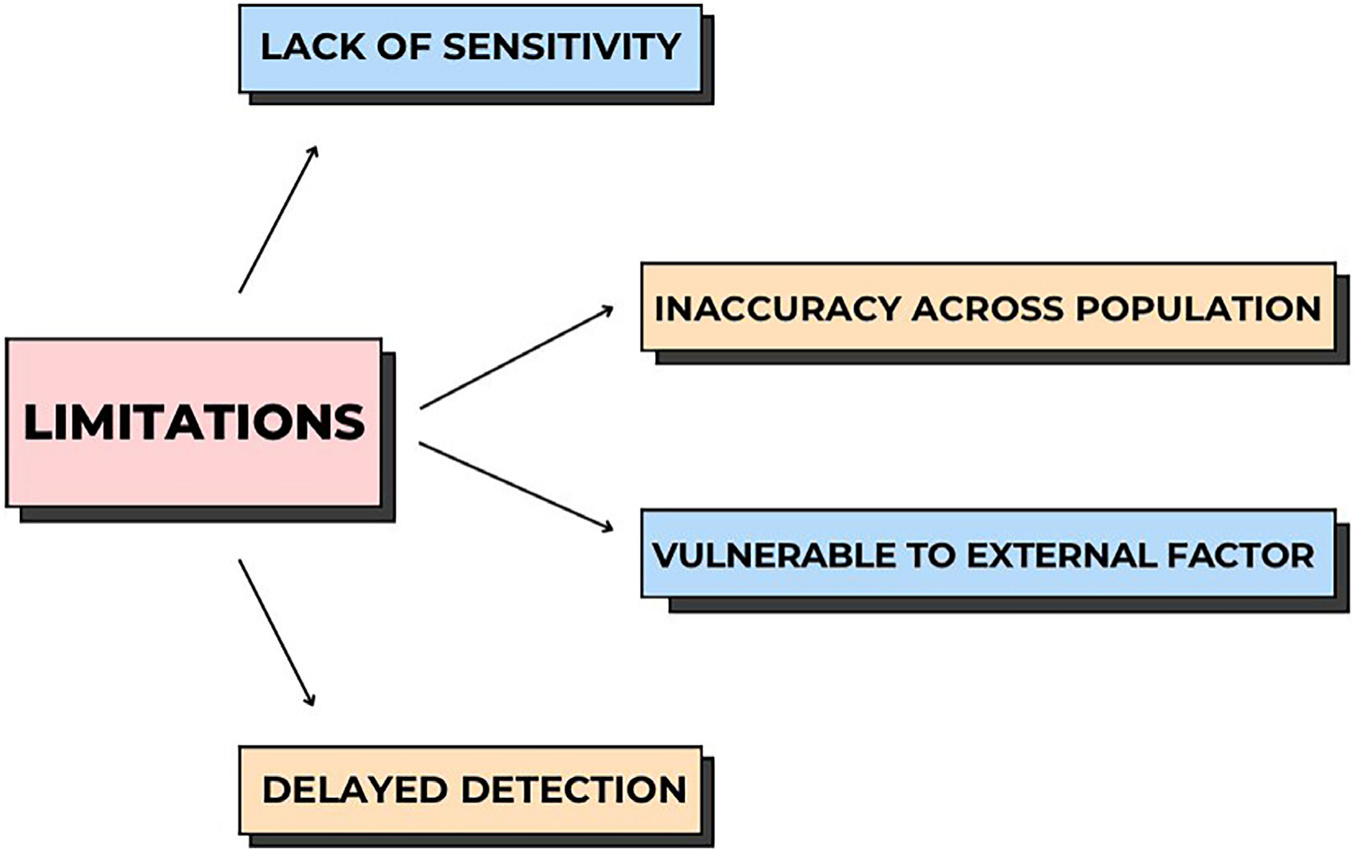
Limitations of traditional approaches.

The challenges mentioned in Figure 2 highlight the need for new biomarkers that provide better disease progression prediction, easier detection, and increased accuracy across individuals. Expanding beyond traditional approaches can improve patient care and CKD diagnosis.

## Categories of Novel Biomarkers for CKD

### Novel Inflammatory and Immune Biomarkers

Since chronic inflammation and immunological dysfunction greatly contribute to the course of CKD, inflammatory and immune indicators are important. These indicators provide a deeper understanding of the disease’s pathways and aid in early kidney damage detection and improved outcome prediction.

#### a) Cystatin C

Cystatin C is produced steadily by all nucleated cells^15^. It is freely filtered by the kidney, undergoes nearly full reabsorption and catabolism in the proximal tubule, and is not significantly excreted in the urine. Therefore, patient variables such gender, age, body size and composition, and nutritional status have a less impact on serum cystatin C levels^16^. However, being an immune-related biomarker, cystatin C levels are altered by inflammatory states and cytokine activity, which can confound the interpretation of this marker in different clinical situations.

Its capacity to identify kidney failure early on, even when creatinine-based estimates are still normal, is one of its main benefits, since it enables early intervention. Beyond kidney function evaluation, studies have also demonstrated that cystatin C is independently linked to cardiovascular disease, ESRD, and all-cause mortality, making it a useful prognostic indicator. Previous studies have reported that the use of cystatin C might be problematic as a marker for renal function, since it can be influenced by non-renal factors such as thyroid disease, corticosteroid administration, and active inflammation^17^.

As there are still variations across laboratory techniques, more standardization and quality assurance should be developed. Furthermore, although cystatin C testing is more costly than creatinine, its potential advantages in early identification, risk classification, and therapy optimization may eventually outweigh the expense^18^.

#### b) IL-8

Interleukin-8 (IL-8) is another novel biomarker that activates neutrophils in inflammation for the immunological response^19^. Numerous cells like macrophages, epithelial cells, and endothelial cells produce IL-8. Due to its function in regulating inflammation and the immune response, IL-8 is typically a useful biomarker for acute kidney injury (AKI). There is a strong correlation between elevated preoperative IL-8 levels and an increased risk of AKI, particularly in children having heart surgery. Studies show that patients with the highest percentile of IL-8 levels are almost five times more likely to suffer AKI than those with the lowest percentile^20,21,22^.

Elevated IL-8 is consistently observed in CKD and dialysis patients, contributing to kidney damage through inflammation, endothelial activation, and fibrosis. The IL-8+781 C/T polymorphism refers to a single nucleotide variation in the IL-8 gene, where the base at position +781 can be either cytosine (C) or thymine (T). Thus, the IL-8+781 C/T polymorphism results in 3 genotypes: CC, TT, and CT. Genetic studies show that the IL-8 +781 T allele appears with a higher frequency in CKD and dialysis patients compared to healthy controls, indicating that the CT and TT genotypes increase inflammatory activity leading to accelerated CKD, serving as a genetic indicator of susceptibility to CKD progression^23^.

In pyelonephritis, IL-8 is also used to distinguish between upper and lower urinary tract infections (UTIs). Serum IL-8 levels are considerably greater in children with pyelonephritis than in those with less severe UTIs. This makes it a valuable marker for early diagnosis and for directing additional diagnostic imaging, such as dimercaptosuccinic acid (DMSA) scans^24^. AKI is mostly linked to IL-8 as a result of its function in immunological activation and inflammation. Its continuous rise, however, might be a sign of ongoing kidney damage and could be useful in identifying AKI patients who are at risk of developing CKD^24,25,26^.

#### c) TNFR1 and TNFR2

The inflammatory processes linked to CKD are significantly influenced by tumor necrosis factor receptors 1 and 2 (TNFR1 and TNFR2). These receptors are useful indicators for diagnosis and prognosis, since their elevated blood levels have been connected to the development of CKD^27^. TNFR1 activation sets off pathways that result in cell death and inflammation. Higher baseline levels of soluble TNFR1 have been linked to a higher risk of CKD progression and ESRD^28^.

TNFR2 is a more promising biomarker for the early diagnosis and progression of CKD. A decrease in kidney function is indicated by rising circulating levels of TNFR2 in early CKD, which are also negatively correlated with eGFR. In contrast to TNFR1, TNFR2 expression increases in kidney tissue when CKD worsens, especially when tubular damage is severe. Hence, TNFR2 has the potential to be a non-invasive diagnostic and predictive tool for CKD management because of its early rise and correlation with CKD severity^29^.

### Oxidative Stress and Endothelial Dysfunction

#### a) ADMA

Asymmetric dimethylarginine (ADMA) is an endogenous inhibitor of nitric oxide synthase, it plays a significant role in endothelial dysfunction, and serves as a novel biomarker^30^. Research has indicated a correlation between decreasing kidney function and the advancement of CKD and increasing plasma ADMA levels.

As an early sign of kidney failure, ADMA levels increase when glomerular filtration rate (GFR) decreases. Higher ADMA levels are associated with a faster rate of disease progression over time, according to research conducted on CKD patients who did not receive dialysis^31^. Due to the kidney’s crucial involvement in ADMA metabolism, poor kidney function causes the compound to accumulate, which exacerbates vascular damage and advances CKD. Monitoring ADMA may therefore be useful for both diagnosis and prognosis, assisting in the stratification of CKD risk and directing early therapies^31,32,33^.

#### b) F2-isoprostanes

F2-isoprostanes are produced in vivo by the peroxidation of arachidonic acid by free radicals. They have a strong correlation with oxidative stress and kidney impairment, making them a novel biomarker for CKD. They are byproducts of lipid peroxidation, and their levels rise in response to increased reactive oxygen species (ROS), which aggravate kidney and vascular damage^34^.

Research has shown that F2-isoprostane levels and eGFR are negatively correlated. Higher F2-isoprostane concentrations are also predictive of a faster CKD progression, which makes them a promising tool for risk stratification and early detection^35^. F2-isoprostanes are useful indicators that go beyond simple detection; they have proven useful in assessing the effectiveness of nephroprotective therapies. It has been demonstrated that statin medication and interventions that target the renin-angiotensin-aldosterone pathway dramatically lower urine levels of 15-F2α-isoprostane in CKD patients, indicating a reduction in oxidative stress^36^.

Measuring F2-isoprostanes provides information on cardiovascular and kidney health since oxidative stress is a major factor in atherosclerosis and endothelial dysfunction, both of which are prevalent in CKD^35^.

### Fibrosis and Tissue Injury Biomarkers

#### a) Kidney Injury Molecule-1 (KIM-1): Predicts tubular injury and CKD progression.

KIM-1 is up-regulated in response to kidney damage making it an early marker of tubular injury and CKD. It is a type 1 transmembrane protein that is normally present in extremely low levels in the kidney, but its expression significantly increases in response to tubular injury. Urine KIM-1 level has been confirmed to be closely related to tissue KIM-1 levels and to correlate with kidney tissue damage. KIM-1 has been demonstrated to be up-regulated in allograft nephropathy as well as several primary and secondary kidney disorders in humans^37,38^.

Elevated KIM-1 levels can indicate the presence of kidney injury, and persistent or rising KIM-1 levels can signal progression to CKD. Urinary KIM-1 levels correlate with the degree of tubular damage, which can be useful for early detection, diagnosis, and monitoring of kidney disease. KIM-1 is involved in kidney inflammation and fibrosis, both of which are key contributors to the progression of CKD^39^.

#### b) Neutrophil Gelatinase-Associated Lipocalin (NGAL): Early indicator of kidney stress.

NGAL is part of the lipocalin family of proteins and an early biomarker for kidney stress. It is primarily expressed in the kidneys, particularly in the proximal tubules, in response to injury. When kidney cells are damaged due to ischemia, toxins, or inflammation, NGAL is upregulated and released into the bloodstream and urine. NGAL levels rise rapidly after injury, unlike creatine. The physiologic effect of NGAL induction in the setting of an injured mature organ, like the kidney, is to significantly preserve function and attenuate apoptosis^40^.

NGAL levels not only signal the presence of kidney injury but also predict the severity of the injury and the risk of progression to more severe kidney conditions. In critically ill patients, timely detection of kidney stress through biomarkers like NGAL can guide early interventions and prevent further complications like multi-organ failure^41^.

#### c) Transforming Growth Factor-β (TGF-β):Key mediator of kidney fibrosis.

TGF-β is a cytokine that plays a pivotal role in kidney injury and fibrosis^40^. TGF-β levels increase significantly in the kidney after injury due to ischemia, toxins, or inflammatory processes. Upon activation, TGF-β binds to its receptors on kidney cells, initiating signaling pathways that promote fibrosis. It drives the transformation of fibroblasts into myofibroblasts and produces excessive extracellular matrix components such as collagen, leading to tissue scarring and kidney dysfunction^42,43^.

In addition to indicating kidney injury, TGF-β is a key mediator that predicts the severity of kidney damage. In response to injury, TGF-β not only induces fibrosis, but also promotes cellular changes such as epithelial-to-mesenchymal transition, which contributes to kidney fibrosis and tubulointerstitial scarring. Hence the increased activation of TGF-β is associated with the progression of kidney disease and worse prognosis^44^.

### Metabolic and Proteomic Biomarkers

#### a) Uromodulin (UMOD):Associated with kidney function preservation.

UMOD, also known as the Tamm-Horsfall protein, helps maintain tubular integrity and protects against CKD progression. Low UMOD levels are associated with an increased risk of CKD, hypertension, and UTIs. Reduced UMOD expression may reflect kidney dysfunction^45^.

This involves the modulation of sodium transport in the kidneys, which directly influences the volume of extracellular fluid and, consequently, blood pressure. Genetic variations can affect kidney sodium handling, contributing to alterations in blood pressure regulation and affect an individual’s overall risk for developing hypertension.

UMOD inhibits the aggregation of calcium oxalate crystals, reducing the likelihood of kidney stone formation. It also plays a role in modulating immune responses and reducing inflammation, which helps protect kidney cells from damage^46^.

#### b) Metabolomics-based biomarkers (e.g., kynurenine-to-tryptophan ratio).

To sustain autoimmunity, tryptophan metabolism through the kynurenine pathway plays a part in controlling the immune response. Tryptophan is an essential amino acid that is used to form proteins and is the precursor to a few bioactive compounds with important physiological functions, such as nicotinamide adenine dinucleotide (NAD+), serotonin, tryptamine, indoles, and kynurenines^47,48^. Tryptophan is absorbed by erythrocytes in the gut and transported into the liver by the hepatic portal system; less than 1% of the tryptophan consumed is used for protein synthesis. Peripheral tissue cells, such as fibroblasts, vascular endothelial cells, and innate immune cells obtained through bloodstream secretion, use the remaining tryptophan. An altered kynurenine-to-tryptophan ratio can indicate disturbances in these processes. This ratio is particularly relevant in conditions like depression, cancer, cardiovascular diseases, and autoimmune disorders. Elevated kynurenine levels, along with decreased tryptophan, are often associated with chronic inflammation and immune dysregulation. As such, monitoring the kynurenine-to-tryptophan ratio can serve as an early biomarker for detecting these conditions^48^.

#### c) Proteomic profiling: Predicting CKD progression via urine protein signatures.

Proteomic profiling is used to track how a patient’s CKD is progressing, providing valuable information to adjust treatment regimens as needed. Urine is an ideal medium for CKD diagnosis because it is easily accessible and contains proteins that directly reflect the state of kidney function. Proteomic profiling of urine can identify changes in protein expression that occur early in the progression of CKD^49^.

Specific protein signatures in urine can indicate various stages of CKD, from early kidney injury to advanced kidney dysfunction. These signatures can help identify patients at elevated risk of rapid progression to ESRD. Some of the key biomarkers identified through proteomic profiling for CKD prediction include NGAL, KIM-1, and albumin^50^.

### Genetic and Epigenetic Biomarkers

#### a) APOL1 risk variants

Recent studies show that APOL1 gene variants have a strong association with focal segmental glomerulosclerosis, HIV-associated nephropathy, hypertensive nephrosclerosis, and lupus nephritis in African Americans. There are striking racial disparities in CKD incidence. African Americans (AA) have a four times greater prevalence of CKD than Caucasians and exhibit a more progressive disease course^51^.

A study by Elliot et al. found that compared with genetic ancestry-matched CKD patients with low-risk APOL1 genotypes, high-risk APOL1 genotype patients were at higher risk of kidney failure (hazard ratio [HR]=1.58), higher eGFR decline (6.55 vs 3.63 mL/min/1.73 m^2^/yr), and were younger at kidney failure (45.1 vs 53.6 years), with the G1/G1 genotype at highest risk. Incidence was lower among patients with CKD with high-risk APOL1 genotypes (2.5%) compared to those with low-risk genotypes (6.7%)^52^. It is inferred in another work by Ekulu et al. that APOL1 risk alleles were prevalent among children living in the Democratic Republic of Congo. HRG carriers are at increased risk of developing early kidney disease, and HIV infection highly increases this risk^53^.

In another research, it was observed that the most significant result, which was composite of ESRD or doubling of serum creatinine level, was seen in 58.1% of the patients in APOL1 high-risk group and 36.6% in APOL1 low-risk group (HR in the high-risk group, 1.88; P < 0.001). The study identified that APOL1 kidney risk variants were associated with higher rates of end-stage kidney disease and progression of chronic kidney disease in black patients relative to white patients, regardless of diabetes status^54,55^.

More recently, podocyte injury has been suspected for the progression of focal segmental glomerulosclerosis (FSGS). Additionally, prolonged podocyte loss in the urine is also seen as a cause factor for CKD and FSGS. Besides these, changes in the lipid profile, damage caused by stress, inflammation, viral infection, hypertension, and alterations in survival and autophagy pathways have also been observed as cause factors for the pathogenesis of tubulointerstitial and glomerular injury leading to CKD. Notably, several mechanisms by which reduced autophagy may affect podocyte function and the pathophysiology of different kidney diseases have been suggested by recent studies. In addition, APOL1 is also recognized in healthy podocytes, and its expression is reduced in FSGS and HIVAN patients. Together, these results suggest that the APOL1 risk alleles may affect the progression of these kidney diseases by modifying the integrity of podocytes. For example, Atg5-deleted mice restricted to podocytes exhibited proteinuria and progressive kidney dysfunction^56^.

#### b) MicroRNAs: potential early markers of kidney damage

Several microRNAs (miRNAs) have a critical role in many cellular and physiological activities such as cell cycle, growth, proliferation, apoptosis, and metabolism. miRNAs are also important in the maintenance of kidney homeostasis and kidney diseases. In vitro and in vivo animal models have shown a critical role of miRNAs in the development of diabetic nephropathy (DN) and in the progression of kidney fibrosis^57^. A substantial body of evidence indicates that miRNAs are involved in the pathophysiology of AKI, CKD, and allograft damage. Different subsets of miRNAs are dysregulated during AKI, CKD, and allograft rejection, which could reflect differences in the physiopathology of these conditions^58^. Preliminary data have shown that miRNA-451 is an early predictor of CKD in diabetic nephropathy. Urinary miR-216a has also been reported to be significantly lower in all patients with type 1 diabetes, with the lowest levels among the microalbuminuria group. Further, positive correlations have been found between urinary miR-377 and albumin-to-creatinine ratio, while urinary miR-216a was negatively correlated to this variable^59^. Table 2 summarizes some of the early biomarkers of CKD.

**Table 2:** Prognosis of CKD by GFR and microalbuminuria degrees^60^

GFR category (mL/min/1.73m^2^)	CKD stage	A1 normal albumin levels (<30 mg/g)	A2 moderate albumin levels (30–300 mg/g)	A3 high albumin levels (>300 mg/g)
≥90	Stage 1	Low risk	Moderate risk	High risk
60–89	Stage 2	Low risk	Moderate risk	High risk
45–59	Stage 3a	Moderate risk	High risk	Very high risk
30–44	Stage 3b	High risk	Very high risk	Very high risk
15–29	Stage 4	Very high risk	Very high risk	Very high risk
< 15	Stage 5	Very high risk	Very high risk	Very high risk

#### c) DNA methylation patterns; epigenetic modifications linked to CKD progression

Epigenetic DNA methylation alterations are involved in the regulation of normal and pathological cellular functions. A disrupted metabolic state, like uremia in CKD, may result in the modification of epigenetics-mediated gene expression. Therefore, uremic memory is established^60,61^. DNA methylation is the key mechanism through which CKD progression is achieved, with regulation of critical genes that are involved in inflammation, fibrosis, and metabolic derangement. Wing et al.^62^ studied African American patients with CKD and observed differential methylation in genes such as RPTOR, an essential component of the mTOR signaling pathway. The study proved that hypermethylation of RPTOR led to reduced expression, which inhibited mTOR function, which is important for cell growth, autophagy, and maintenance of energy balance.

Chu et al.^63^ further performed an epigenomewide association study (EWAS) and found Klotho gene hypermethylation, a well-known renoprotective factor. Klotho downregulation resulting from epigenetic silencing was linked with premature kidney function deterioration and increased oxidative stress, vascular calcification, and fibrosis, ultimately leading to worsening CKD outcomes. In addition, Ko et al.^64^ investigated genome-wide methylation status and detected extensive global DNA hypomethylation in CKD patients, especially in inflammation-related genes such as NF-κB pathway genes. This epigenetic hypomethylation promoted pro-inflammatory cytokine upregulation, further provoking immune dysfunction and chronic inflammation in CKD. Collectively, these data indicate that epigenetic modifications server not only as disease progression biomarkers but also as therapeutic targets too.

## Clinical Applications and Future Directions

The future of CKD management lies in the identification and use of new biomarkers besides the traditional serum creatinine and eGFR. New biomarkers such as urinary NGAL and KIM-1 have shown potential in identifying kidney injury before overt functional impairment. In clinical studies, performing well above the baseline, demonstrated that urinary NGAL levels >150 ng/mL indicated injury and AKI-to-CKD transition with high sensitivity, while KIM-1 has been consistent at 95% specificity across diabetic nephropathy cohorts^65,66^. Soluble urokinase plasminogen activator receptor (suPAR) has also been identified as a marker of systemic inflammation and CKD progression that offers insight into disease processes beyond kidney-specific injury. In multivariate adjusted analyses, elevated suPAR (>3,040 pg/mL) independently predicted more rapid loss of eGFR (highest vs. lowest quartile: -4.2 versus -0.9 mL/min/1.73 m^2^ per year). Five-year rates of CKD occurred in 41% for high-suPAR patients and in 12% among low-suPAR groups^67^. Omics technologies, particularly proteomics, metabolomics, and epigenomics, continue to uncover increasingly sophisticated molecular signatures linked to CKD risk.

DNA methylation patterns, circulating microRNAs, and metabolite profiles are some promising non-invasive candidates that have arisen for risk stratification, discriminating between high-risk versus low-risk patients for CKD progression. Translating these biomarkers into clinical practice, particularly with the aid of artificial intelligence and machine learning algorithms, would allow for highly individualized risk determination, with the potential for earlier intervention by nephrologists and tailoring of therapy based on each patient’s individual disease signature.

Furthermore, biomarker-based treatment approaches where treatment is based on specific molecular profiles are likely to usher in a new dawn of personalized medicine in nephrology. By shifting from a reactive to a proactive management, emerging biomarkers can transform CKD management, improve early detection, retard disease progression, and ultimately reduce the burden of ESRD incidence.

## Conclusion

Novel biomarkers for CKD risk stratification have the potential to revolutionize patient care by enabling early diagnosis, more precise prognosis, and personalized treatment approaches. Although promising, further large-scale validation studies are needed to establish their routine clinical use. Multidisciplinary incorporation of these biomarkers into existing clinical frameworks would unlock the full potential of CKD management and patient benefit.

## Data Availability

Data sharing is not applicable as all of the information synthesized in this review is available online.
