# Supplementary material for: Novel biomarkers for CKD risk stratification: a literature review
Source: J Bras Nefrol. 2026 Jan 9;48(1):e20250109. doi: 10.1590/2175-8239-JBN-2025-0109en (PMC12788398; doi:10.1590/2175-8239-JBN-2025-0109en)
Supplement: Translation

# Translation: Novos biomarcadores para estratificação do risco de DRC: uma revisão da literatura

**DOI:** 10.1590/2175-8239-JBN-2025-0109pt

**Authors:** Sariya Khan, Aleena Zobairi, Elaf Rehan, Ashraf Hussein Mohammed

**Affiliations:** 1 Batterjee Medical College, General Medicine Practice Program, Jeddah, Arábia Saudita.; 2 Mansoura University, Mansoura, Egito.; 3 Saudi German Hospital, Nephrology Department, Jeddah, Arábia Saudita.

**Keywords:** Insuficiência Renal Crônica, Bioindicador, Biomarcadores

## Abstract

**Introdução:**

Doença renal crônica (DRC) é uma doença progressiva, com elevada morbimortalidade, demandando estratificação precoce e precisa do risco para um manejo ideal. Biomarcadores tradicionais - creatinina sérica, taxa de filtração glomerular estimada (TFGe) - são insuficientes para detecção precoce da DRC e do prognóstico em longo prazo. Novos biomarcadores surgiram como ferramentas eficazes para complementar o diagnóstico, prognóstico e monitoramento terapêutico da DRC.

**Objetivo:**

Determinar o potencial de novos biomarcadores na estratificação do risco de DRC e sua significância clínica para aprimorar a detecção precoce, monitorar a progressão da doença e desenvolver estratégias terapêutica individualizadas.

**Métodos:**

Realizou-se revisão da literatura por meio de buscas nas bases de dados *PubMed*, *Scopus* e *Embase* para identificar estudos sobre novos biomarcadores da DRC, incluindo cistatina C, lipocalina associada à gelatinase neutrofílica (NGAL), molécula de lesão renal 1 (KIM-1) e microRNAs específicos.

**Resultados:**

Evidências emergentes sugerem que novos biomarcadores oferecem capacidades preditivas superiores comparados aos biomarcadores tradicionais. A cistatina C demonstra maior precisão na estimativa da função renal, enquanto NGAL e KIM-1 são biomarcadores de lesão renal precoce. MicroRNAs apresentam potencial para distinguir entre subtipos de DRC e predizer progressão da doença. A aplicação clínica desses biomarcadores pode aprimorar a estratificação do risco de DRC, permitindo estratégias de intervenção mais direcionadas.

**Conclusão:**

Novos biomarcadores na estratificação do risco de DRC representam um marco na nefrologia, oferecendo detecção precoce aprimorada e maior precisão no prognóstico. Embora promissores, são necessários estudos adicionais em larga escala e validação clínica antes que possam ser utilizados rotineiramente.

## Introdução

Uma das principais causas de morbidade e mortalidade por doenças não transmissíveis é a doença renal crônica (DRC). A DRC é uma condição de longo prazo na qual as unidades responsáveis pela filtração dos rins (néfrons) perdem gradualmente a capacidade de excretar produtos residuais e regular processos metabólicos importantes, levando ao acúmulo de toxinas, retenção de líquidos e desequilíbrios em eletrólitos como o potássio e o sódio. É definida por uma taxa de filtração glomerular (TFGe) inferior a 60 mL/min/1,73 m^2^ ou pela presença de lesão renal (por exemplo, proteinúria) por um período superior a três meses^1^. As principais causas de DRC incluem diabetes mellitus, hipertensão e glomerulonefrite, com fatores de risco como envelhecimento, obesidade, tabagismo e predisposição genética contribuindo para sua progressão. Sem intervenção oportuna, a DRC pode progredir para doença renal em estágio terminal (DRET), exigindo diálise ou transplante renal para a sobrevida^2^.

A DRC é uma das principais causas de óbito e sofrimento no século XXI. Sua prevalência vem aumentando globalmente, sendo atualmente o sétimo principal fator de risco para mortalidade em todo o mundo, com previsão de atingir a 5ª posição. É uma das poucas doenças não transmissíveis que apresentou um aumento no número de óbitos associados nas últimas duas décadas^3,4^. A descrição dos diferentes estágios da DRC está resumida na Tabela 1. A DRC é uma doença degenerativa que afeta cerca de 800 milhões de pessoas em todo o mundo, o que representa mais de 10% da população global. Estima-se que 80% do ônus global ocorra em países de baixa e média renda (PBMR), com 25% dos casos acometendo indivíduos com menos de 60 anos de idade. A DRC representa um ônus substancial nessas regiões, que muitas vezes não dispõem de recursos adequados para o manejo de suas consequências. Em contrapartida, os países de alta renda normalmente destinam de 2 a 3% de seus orçamentos anuais de saúde ao tratamento da doença renal em estágio terminal, apesar de esses pacientes representarem menos de 0,03% da população total^5^.

**Tabela 1:** Descrição dos estágios da DRC^3,4^

Estágio da DRC	Características	Taxa de filtração glomerular (TFGe) (mL/min/1,73 m^2^)
Estágio 1	Função renal normal, mas com alguns sinais de lesão renal, como presença de proteína na urina.	≥ 90
Estágio 2	Função renal levemente reduzida	60–89
Estágio 3b	Redução moderada da função renal	30–44
Estágio 3a	Redução moderada da função renal	45–59
Estágio 4	Redução grave da função renal	15–29
Estágio 5	Insuficiência renal	< 15

A DRC impõe um ônus econômico e social significativo mundialmente, especialmente em PBMR, devido aos custos elevados de diálise e transplante. Muitos pacientes nesses países não têm acesso a cuidados médicos de baixo custo, o que resulta em altas taxas de mortalidade entre aqueles com DRC que não podem arcar com os custos de tratamentos que salvam vidas^5^.

A detecção precoce e a estratificação do risco são fundamentais no manejo da DRC, afetando significativamente a progressão da doença e os desfechos dos pacientes. A identificação da DRC em seus estágios iniciais permite uma intervenção oportuna, que pode retardar ou mesmo interromper sua progressão para estágios mais graves. Também melhora o prognóstico ao possibilitar um manejo mais adequado das condições associadas, como hipertensão e diabetes, o que reduz o risco de complicações, incluindo doenças cardiovasculares. Ao abordar precocemente essas condições subjacentes, os profissionais de saúde podem ajudar a reduzir o risco de complicações, incluindodoenças cardiovasculares, acidente vascular cerebral e insuficiência cardíaca - condições mais comuns em pacientes com DRC e que contribuem para o aumento da morbimortalidade^6,7^. Além disso, o aspecto financeiro da intervenção precoce é significativo para o tratamento da DRC em seus estágios iniciais, uma vez que essa abordagem não apenas se mostra mais eficaz, como também consideravelmente mais econômicado que o manejo dos estágios avançados da doença^7^. Os diferentes estágios da DRC e da RAC estão descritos na Figura 1.

**Figura 1.** Os diferentes estágios da DRC e da RAC^11^.

Considere a utilização de eGFRcistatinaC para pessoas com DRC G3aA1 (ver recomendações KDIGO 1.1.14 e 1.1.15).

Abreviaturas – ACR: relação albumina/creatinina; DRC: doença renal crónica; TFG: taxa de filtração glomerular. Adaptado com autorização de Kidney Disease: Improving Global Outcomes (KDIGO) CKD Work Group (2013) KDIGO 2012 clinical practice guideline for the evaluation and management of chronic kidney disease. Kidney International (Supl. 3): 1–150.

Biomarcadores tradicionais como creatinina sérica, TFGe e proteinúria têm sido essenciais no diagnóstico e monitoramento da DRC. No entanto, eles apresentam diversas limitações que podem ter impacto na eficácia da detecção precoce, do diagnóstico e do manejo^8,9,10^.

O objetivo desta revisão é explorar e avaliar o potencial de novos biomarcadores para aprimorar a estratificação de risco na DRC. São avaliados biomarcadores emergentes em diversas categorias, incluindo avaliação de risco e monitoramento da progressão da DRC. Ao concentrar-se na utilidade clínica e nos desafios desses novos biomarcadores, a revisão busca fornecer *insights* sobre como eles podem revolucionar o manejo da DRC e contribuir para otimizar estratégias de tratamento personalizadas. Os ensaios clínicos em andamento têm como foco a validação de novos biomarcadores para avaliação do risco de DRC, a melhoria da detecção precoce e a integração dos biomarcadores na prática clínica, enquanto pesquisas futuras devem envolver padronização, estudos de longo prazo e avaliações de custo-efetividade para facilitar a ampla adoção.

## Métodos

Este artigo é uma revisão narrativa que sintetiza novas evidências sobre a predição precoce da DRC utilizando biomarcadores novos e atualizados. Foi realizada uma busca bibliográfica nas bases de dados *PubMed/* MEDLINE, *Web of Science* e *Google Scholar*, desde a criação das bases até fevereiro de 2025. As palavras-chave de maior importância foram, em inglês, “*CKD biomarkers*”, “*cystatin C*”, “*neutrophil gelatinase-associated lipocalin* (NGAL)”, “*kidney injury molecule-1* (KIM-1)” e “*microRNAs*”. Operadores booleanos (AND/OR) e truncamentos foram aplicados quando relevante. Foram considerados estudos publicados em inglês e que abordassem novos biomarcadores na DRC. Incluíram-se artigos originais (caso-controle, coorte, transversal e série de casos), revisões sistemáticas e revisões narrativas. Por se tratar de uma revisão narrativa, não foi aplicado nenhum processo formal de triagem, inclusão ou exclusão. Os artigos foram selecionados com base em sua relevância para os objetivos da revisão e no julgamento dos autores, a partir de buscas em bancos de dados e da literatura previamente conhecida. Não foram impostas restrições quanto ao desenho do estudo ou ano de publicação, e a inclusão das fontes foi determinada por sua contribuição percebida para a síntese temática, em vez da adesão a critérios de elegibilidade predefinidos.

## Biomarcadores Tradicionais vs. Novos na Estratificação do Risco de DRC

O diagnóstico e o monitoramento da DRC têm se beneficiado significativamente dos biomarcadores tradicionais, como albumina urinária, creatinina sérica e TFGe. No entanto, esses biomarcadores apresentam uma série de desvantagens que podem comprometer sua capacidade de detectar precocemente a doença, avaliá-la com precisão e predizer seu prognóstico^11,12^.

A creatinina sérica é um biomarcador tradicional bastante comum. Sua dependência da massa muscular torna-a um indicador pouco confiável da função renal, podendo levar a classificações incorretas em indivíduos com massa muscular excepcionalmente alta ou baixa. Além disso, não é adequada para a identificação precoce da DRC, já que seus níveis só se elevam após a ocorrência de danos renais substanciais.Seus níveis são ainda mais afetados por fatores não renais, como dieta, medicamentos e hidratação^13,14^.

De forma semelhante, a TFGe, que se baseia na creatinina sérica, apresenta desvantagens, como a sensibilidade a variáveis não renais e à massa muscular. As diferenças de idade e etnia também podem resultar em classificações incorretas. Além disso, a TFGe não é um indicador confiável para indivíduos com padrões alimentares atípicos, metabolismo alterado ou tamanho corporal extremo. A albumina urinária também é utilizada, mas pode ser influenciada por variáveis temporárias, como doenças e atividade física, o que pode resultar em falsos positivos. Ela não detecta lesões intersticiais ou tubulares e identifica, principalmente, danos glomerulares. Além disso, a presença de albuminúria não prediz necessariamente a DRC, e alguns indivíduos podem apresentar progressão da doença, mesmo na ausência de albuminúria^13,14^.

Os desafios mencionados na Figura 2 destacam a necessidade de novos biomarcadores que ofereçam melhor capacidade de predição da progressão da doença, detecção facilitada e maior precisão entre os indivíduos. A expansão além das abordagens tradicionais pode aprimorar o cuidado ao paciente e o diagnóstico da DRC.

**Figura 2.** Limitações das abordagens tradicionais.

## Categorias de Novos Biomarcadores Para DRC

### Novos Biomarcadores Inflamatórios e Imunológicos

Como a inflamação crônica e a disfunção imunológica contribuem significativamente para o curso da DRC,os indicadores inflamatórios e imunológicos são importantes. Esses indicadores proporcionam uma compreensão mais abrangente das vias da doença e auxiliam na detecção precoce de lesões renais e na melhoria da predição de desfechos.

#### a) Cistatina C

A cistatina C é produzida de forma constante por todas as células nucleadas^15^. É livremente filtrada pelos rins, sofre reabsorção e catabolismo quase completos no túbulo proximal e não é excretada em quantidades significativas na urina. Portanto, variáveis do paciente, como sexo, idade, tamanho e composição corporais, além do estado nutricional, exercem menor impacto sobre os níveis séricos de cistatina C^16^. No entanto, por ser um biomarcador relacionado à resposta imune, os níveis de cistatina C são alterados por estados inflamatórios e pela atividade das citocinas, o que pode confundir a interpretação desse biomarcador em diferentes situações clínicas.

Sua capacidade de identificar precocemente a insuficiência renal, mesmo quando as estimativas baseadas em creatinina ainda se encontram dentro da normalidade, é um dos seus principais benefícios, uma vez que permite uma intervenção precoce. Além da avaliação da função renal, estudos também demonstraram que a cistatina C está independentemente associada a doenças cardiovasculares, DRET e mortalidade por todas as causas, tornando-a um indicador prognóstico útil. Estudos anteriores relataram que o uso da cistatina C como biomarcador da função renal pode ser problemático, uma vez que seus níveis podem ser influenciados por fatores não renais, como doenças da tireoide, administração de corticosteroides e inflamação ativa^17^.

Como ainda existem variações entre as técnicas laboratoriais, é necessário desenvolver uma maior padronização e garantia de qualidade. Além disso, embora o teste da cistatina C seja mais caro do que o da creatinina, suas potenciais vantagens na identificação precoce, classificação de risco e otimização da terapia podem, eventualmente, compensar o custo^18^.

#### b) IL-8

A interleucina-8 (IL-8) é outro novo biomarcador que atua na ativação de neutrófilos no processo inflamatório para a resposta imune^19^. Diversas células, como macrófagos, células epiteliais e células endoteliais, produzem IL-8. Devido à sua função na regulação da inflamação e da resposta imune, a IL-8 é tipicamente um biomarcador útil para a injúria renal aguda(IRA). Existe uma forte correlação entre níveis elevados de IL-8 no pré-operatório e um risco aumentado de IRA, particularmente em crianças submetidas à cirurgia cardíaca. Estudos demonstram que pacientes com os percentis mais elevados de IL-8 apresentam uma probabilidade quase cinco vezes maior de apresentar IRA do que aqueles com os percentis mais baixos^20,21,22^.

Níveis elevados de IL-8 são consistentemente observados em pacientes com DRC e em diálise, contribuindo para danos renais por meio de inflamação, ativação endotelial e fibrose. O polimorfismo IL-8+781 C/T refere-se a uma variação de um único nucleotídeo no gene IL-8, em que a base na posição +781 pode ser citosina (C) ou timina (T). Assim, o polimorfismo IL-8+781 C/T resulta em três genótipos: CC, TT e CT. Estudos genéticos demonstram que o alelo T do polimorfismo IL-8 +781 T aparece com maior frequência em pacientes com DRC e em diálise quando comparados a controles saudáveis, indicando que os genótipos CT e TT aumentam a atividade inflamatória, levando à aceleração da DRC e servindo como um indicador genético de suscetibilidade à progressão da doença^23^.

Na pielonefrite, a IL-8 também é utilizada para distinguir entre infecções do trato urinário (ITU) superior e inferior. Os níveis séricos de IL-8 são consideravelmente mais elevados em crianças com pielonefrite do que naquelas com ITU menos graves. Isso a torna um biomarcador valioso para o diagnóstico precoce e para orientar exames de imagem diagnósticos adicionais, como a cintilografia com ácido dimercaptossuccínico (DMSA)^24^. A IRA está principalmente associada à IL-8 em decorrência de sua função na ativação imunológica e na inflamação. Seu aumento contínuo, no entanto, pode indicar danos renais em andamento e pode ser útil na identificação de pacientes com IRA que estejam em risco de desenvolver DRC^24,25,26^.

#### c) TNFR1 e TNFR2

Os processos inflamatórios associados à DRC são significativamente influenciados pelos receptores do fator de necrose tumoral 1 e 2 (TNFR1 e TNFR2). Esses receptores são indicadores úteis para diagnóstico e prognóstico, uma vez que seus níveis elevados no sangue têm sido associados ao desenvolvimento da DRC^27^. A ativação do TNFR1 desencadeia vias que resultam em morte celular e inflamação. Níveis basais mais elevados de TNFR1 solúvel têm sido associados a um risco maior de progressão da DRC e de DRET^28^.

O TNFR2 é um biomarcador mais promissor para o diagnóstico precoce e a progressão da DRC. Uma diminuição na função renal é indicada pelo aumento dos níveis circulantes de TNFR2 na DRC precoce, que também estão negativamente correlacionados à TFGe. Em contraste com o TNFR1, a expressão do TNFR2 aumenta no tecido renal à medida que a DRC se agrava, especialmente quando o dano tubular é grave. Assim, oTNFR2 tem potencial para atuar como uma ferramenta diagnóstica e preditiva não invasiva para o manejo da DRC, devido ao seu aumento precoce e à sua correlação com a gravidade da doença^29^.

### Estresse Oxidativo e Disfunção Endotelial

#### a) ADMA

A dimetilarginina assimétrica (ADMA) é um inibidor endógeno da óxido nítrico sintase. Ela desempenha um papel significativo na disfunção endotelial e serve como um novo biomarcador^30^. Pesquisas têm indicado uma correlação entre a redução da função renal e o avanço da DRC, bem como o aumento dos níveis plasmáticos de ADMA.

Como um sinal precoce de insuficiência renal, os níveis de ADMA aumentam à medida que a taxa de filtração glomerular (TFG) diminui. Segundo pesquisas realizadas em pacientes com DRC que não receberam diálise, níveis mais elevados de ADMA estão associados a uma velocidade mais rápida de progressão da doença ao longo do tempo^31^. Devido ao envolvimento crucial dos rins no metabolismo da ADMA, a função renal deficiente causa o acúmulo do composto, o que agrava os danos vasculares e promove o avanço da DRC. O monitoramento da ADMA pode, portanto, ser útil tanto para o diagnóstico quanto para o prognóstico, auxiliando na estratificação do risco de DRC e no direcionamento de terapias precoces^31,32,33^.

#### b) F2-isoprostanos

Os F2-isoprostanos são produzidos in vivo pela peroxidação do ácido araquidônico por radicais livres. Apresentam uma forte correlação com o estresse oxidativo e a insuficiência renal, tornando-os um novo biomarcador para a DRC. São subprodutos da peroxidação lipídica, e seus níveis aumentam em resposta à elevação das espécies reativas de oxigênio (ERO), que agravam o dano renal e vascular^34^.

Pesquisas demonstraram que os níveis de F2-isoprostano e a TFGe estão negativamente correlacionados. Concentrações mais elevadas de F2-isoprostano também são preditivas de uma progressão mais rápida da DRC, o que os torna uma ferramenta promissora para a estratificação de risco e detecção precoce^35^. Os F2-isoprostanos são indicadores úteis que vão além da simples detecção; eles se mostraram úteis na avaliação da eficácia de terapias nefroprotetoras. Foi demonstrado que medicamentos à base de estatina e intervenções direcionadas à via renina-angiotensina-aldosterona reduzem drasticamente os níveis urinários de 15-F2α-isoprostano em pacientes com DRC, indicando uma redução do estresse oxidativo^36^.

A medição dos F2-isoprostanos fornece informações sobre a saúde cardiovascular e renal, uma vez que o estresse oxidativo é um fator importante na aterosclerose e na disfunção endotelial, ambas prevalentes na DRC^35^.

### Biomarcadores de Fibrose e Lesão Tecidual

#### a) Molécula de lesão renal-1 (KIM-1):Prediz lesão tubular e progressão da DRC.

A KIM-1 é regulada positivamente em resposta ao dano renal, o que a torna um marcador precoce de lesão tubular e DRC. É uma proteína transmembrana do tipo 1, normalmente presente em níveis extremamente baixos nos rins, mas cuja expressão aumenta significativamente em resposta à lesão tubular. Foi confirmado que os níveis de KIM-1 na urina estão intimamente relacionados aos níveis dessa molécula nos tecidos e correlacionam-se ao dano no tecido renal. Demonstrou-se que a KIM-1 é regulada positivamente nos casos de nefropatia do aloenxerto e em diversas doenças renais primárias e secundárias em seres humanos^37,38^.

Níveis elevados de KIM-1 podem indicar a presença de injúria renal, e níveis persistentes ou crescentes dessa molécula podem sinalizar a progressão para DRC. Os níveis urinários de KIM-1 estão correlacionados com o grau de dano tubular, o que pode ser útil para a detecção precoce, o diagnóstico e o monitoramento da doença renal. A KIM-1 está envolvida nos processos de inflamação e fibrose renal, ambos fatores-chave para a progressão da DRC^39^.

#### b) Lipocalina associada à gelatinase neutrofílica (NGAL): Indicador precoce de estresse renal.

A NGAL é uma proteína que pertence à família das lipocalinas e constitui um biomarcador precoce de estresse renal. É expressa principalmente nos rins, particularmente nos túbulos proximais, em resposta a lesões. Quando as células renais são danificadas por isquemia, toxinas ou inflamação, a NGAL é regulada positivamente e liberada na corrente sanguínea e na urina. Os níveis de NGAL aumentam rapidamente após uma lesão, diferentemente da creatina. O efeito fisiológico da indução de NGAL no contexto de um órgão maduro lesionado, como o rim, é preservar significativamente sua função e atenuar a apoptose^40^.

Os níveis dessa proteína não apenas sinalizam a presença de injúria renal, mas também predizem a gravidade da lesão e o risco de progressão para condições renais mais graves. Em pacientes gravemente enfermos, a detecção imediata do estresse renal por meio de biomarcadores como a NGAL pode orientar intervenções precoces e prevenir complicações adicionais, como a falência múltipla de órgãos^41^.

#### c) Fator de Crescimento Transformadorβ(TGF-β):Principal mediador da fibrose renal.

O TGF-β é uma citocina que desempenha um papel fundamental na injúria e na fibrose renal^40^. Seus níveis aumentam significativamente nos rins após lesões causadas por isquemia, toxinas ou processos inflamatórios. Quando ativado, o TGF-β se liga aos seus receptores nas células renais, iniciando vias de sinalização que promovem a fibrose. Induz a transformação de fibroblastos em miofibroblastos e promove a produção excessiva de componentes da matriz extracelular, como o colágeno, levando à cicatrização do tecido e à disfunção renal^42,43^.

Além de indicar injúria renal, o TGF-β é um mediador fundamental que prediz a gravidade do dano renal. Em resposta à lesão, o TGF-β não apenas induz a fibrose, mas também promove alterações celulares, como a transição epitelial-mesenquimal, que contribui para a fibrose renal e a formação de cicatrizes tubulointersticiais. Dessa forma, o aumento da ativação do TGF-β está associado à progressão da doença renal e a um pior prognóstico^44^.

### Biomarcadores Metabólicos e Proteômicos

#### a) Uromodulina (UMOD):Associada à preservação da função renal.

A UMOD, também conhecida como proteína *Tamm-Horsfall*, contribui para a manutenção da integridade tubular e protege contra a progressão da DRC. Níveis reduzidos de UMOD estão associados a um risco aumentado de DRC, hipertensão e ITU. A redução da expressão de UMOD pode refletir disfunção renal^45^.

Esse processo envolve a modulação do transporte de sódio nos rins, o que influencia diretamente o volume de fluido extracelular e, consequentemente, a pressão arterial. Variações genéticas podem afetar o metabolismo do sódio nos rins, contribuindo para alterações na regulação da pressão arterial e impactando o risco geral de um indivíduo desenvolver hipertensão.

A UMOD inibe a agregação de cristais de oxalato de cálcio, reduzindo a probabilidade de formação de cálculos renais. Também desempenha um papel na modulação das respostas imunes e na redução da inflamação, o que contribui para proteger as células renais contra danos^46^.

#### b) Biomarcadores com base na metabolômica (por exemplo, relação quinurenina/triptofano).

Para sustentar a autoimunidade, o metabolismo do triptofano através da via da quinurenina desempenha um papel no controle da resposta imune. O triptofano é um aminoácido essencial utilizado na formação de proteínas e é o precursor de alguns compostos bioativos com funções fisiológicas importantes, tais como a nicotinamida adenina dinucleotídeo (NAD+), a serotonina, a triptamina, os indóis e as quinureninas^47,48^. O triptofano é absorvido por eritrócitos no intestino e transportado para o fígado pelo sistema porta hepático; menos de 1% do triptofano consumido é utilizado para a síntese proteica. As células dos tecidos periféricos, como fibroblastos, células endoteliais vasculares e células da imunidade inata, obtidas por meio de secreção na corrente sanguínea, utilizam o triptofano restante. Uma relação quinurenina/triptofano alterada pode indicar distúrbios nesses processos. Essa relação é particularmente relevante em condições como depressão, câncer, doenças cardiovasculares e distúrbios autoimunes. Níveis elevados de quinurenina, juntamente com a redução do triptofano, estão frequentemente associados à inflamação crônica e à desregulação imunológica. Assim, o monitoramento da relação quinurenina/triptofano pode funcionar como um biomarcador precoce para a detecção dessas condições^48^.

#### c) Perfil proteômico:Predição da progressão da DRC por assinaturas proteicas urinárias.

O perfil proteômico é utilizado para monitorar a progressão da DRC em um paciente, fornecendo informações valiosas para o ajuste dos regimes de tratamento conforme necessário. A urina é um meio ideal para o diagnóstico da DRC, pois é de fácil obtenção e contém proteínas que refletem diretamente o estado da função renal. O perfil proteômico da urina pode identificar alterações na expressão proteica que ocorrem precocemente na progressão da DRC^49^.

Assinaturas proteicas específicas na urina podem indicar diversos estágios da DRC, desde injúria renal precoce até disfunção renal avançada. Essas assinaturas podem auxiliar na identificação de pacientes com risco elevado de progressão rápida para DRET. Alguns dos principais biomarcadores identificados por meio do perfil proteômico para a predição da DRC incluem NGAL, KIM-1 e albumina^50^.

### Biomarcadores Genéticos e Epigenéticos

#### a) Variantes de risco APOL1

Estudos recentes demonstram que as variantes do gene APOL1 apresentam uma forte associação com glomeruloesclerose segmentar e focal, nefropatia associada ao HIV, nefroesclerose hipertensiva e nefrite lúpica em afro-americanos. Existem disparidades raciais notáveis na incidência de DRC: os afroamericanos (AA) apresentam uma prevalência quatro vezes maior de DRC em comparação aos caucasianos e exibem um curso mais progressivo da doença^51^.

Um estudo realizado por Elliot et al. constatou que, em comparação aos pacientes com DRC com genótipos APOL1 de baixo risco e ascendência genética compatível, aqueles com genótipos APOL1 de alto risco apresentaram maior risco de insuficiência renal (*hazard ratio* [HR] = 1,58), maior declínio da TFGe (6,55 vs. 3,63 mL/min/1.73 m^2^/ano), e apresentaram menor idade na ocorrência de insuficiência renal (45,1 vs. 53,6 anos), sendo o genótipo G1/G1 o de maior risco. A incidência foi menor entre pacientes com DRC com genótipos APOL1 de alto risco (2,5%) em comparação àqueles com genótipos de baixo risco (6,7%)^52^. Outro estudo, realizado por Ekulu et al., inferiu que os alelos de risco do APOL1 foram prevalentes entre crianças que vivem na República Democrática do Congo. Portadores de genótipos de alto risco apresentam maior probabilidade de desenvolver doença renal precoce, e a infecção pelo HIV aumenta significativamente esse risco^53^.

Em outra pesquisa, observou-se que o resultado mais significativo - composto por DRET ou duplicação do nível de creatinina sérica - foi observado em 58,1% dos pacientes no grupo APOL1 de alto risco e em 36,6% no grupo de baixo risco (HR no grupo de alto risco, 1,88; P < 0,001). O estudo identificou que as variantes de risco renal do APOL1 estiveram associadas a taxas mais elevadas de doença renal em estágio terminal e progressão da doença renal crônica em pacientes negros em relação aos pacientes brancos, independentemente do status de diabetes^54,55^.

Mais recentemente, suspeita-se que a lesão podocitária seja responsável pela progressão da glomeruloesclerose segmentar e focal (GESF). Além disso, a perda prolongada de podócitos na urina também é considerada um fator causal para DRC e GESF. Além desses fatores, alterações no perfil lipídico, danos causados pelo estresse, inflamação, infecção viral, hipertensão e modificações na sobrevida e nas vias de autofagia também foram observadas como fatores causais para a patogênese da lesão tubulointersticial e glomerular que leva à DRC. Notavelmente, estudos recentes sugeriram diversos mecanismos pelos quais a redução da autofagia pode afetar a função dos podócitos e a fisiopatologia de diferentes doenças renais. Além disso, o APOL1 também é reconhecido em podócitos saudáveis, e sua expressão é reduzida em pacientes com GESF e HIVAN. Em conjunto, esses resultados sugerem que os alelos de risco do APOL1 podem influenciar a progressão dessas doenças renais ao modificar a integridade dos podócitos. Por exemplo, camundongos com deleção do Atg5 restrita aos podócitos apresentaram proteinúria e disfunção renal progressiva^56^.

#### b) MicroRNAs: potenciais biomarcadores precoces de lesão renal

Diversos microRNAs (miRNAs) desempenham um papel fundamental em inúmeras atividades celulares e fisiológicas, como ciclo celular, crescimento, proliferação, apoptose e metabolismo. Os miRNAs também são importantes na manutenção da homeostase renal e nas doenças renais. Modelos animais *in vitro* e *in vivo* demonstraram a importância dos miRNAs no desenvolvimento da nefropatia diabética (ND) e na progressão da fibrose renal^57^. Um conjunto substancial de evidências indica que os miRNAs estão envolvidos na fisiopatologia da IRA, DRC e danos ao aloenxerto. Diferentes subconjuntos de miRNAs apresentam desregulação durante a IRA, a DRC e a rejeição do aloenxerto, o que pode refletir diferenças na fisiopatologia dessas condições^58^. Dados preliminares demonstraram que o miRNA-451 é um preditor precoce de DRC na nefropatia diabética. Também foi relatado que o miR-216a urinário esteve significativamente mais baixo em todos os pacientes com diabetes tipo 1, apresentando os níveis mais baixos entre aqueles do grupo com microalbuminúria. Além disso, foram encontradas correlações positivas entre o miR-377 urinário e a relação albumina/creatinina, enquanto o miR-216a urinário apresentou correlação negativa com essa variável^59^. A Tabela 2 resume alguns dos biomarcadores precoces de DRC.

**Tabela 2:** Prognóstico da DRC de acordo com TFG e graus de microalbuminúria^60^

Categoria da TFG (mL/min/1,73m^2^)	Estágio da DRC	A1 Níveis normais de albumina (<30 mg/g)	A2 Níveis moderados de albumina (30–300 mg/g)	A3 Níveis elevados de albumina (>300 mg/g)
≥ 90	Estágio 1	Risco baixo	Risco moderado	Risco elevado
60–89	Estágio 2	Risco baixo	Risco moderado	Risco elevado
45–59	Estágio 3a	Risco moderado	Risco elevado	Risco muito elevado
30–44	Estágio 3b	Risco elevado	Risco muito elevado	Risco muito elevado
15–29	Estágio 4	Risco muito elevado	Risco muito elevado	Risco muito elevado
< 15	Estágio 5	Risco muito elevado	Risco muito elevado	Risco muito elevado

#### c) Padrões de metilação do DNA; modificações epigenéticas associadas à progressão da DRC

Alterações epigenéticas da metilação do DNA estão envolvidas na regulação das funções celulares normais e patológicas. Um estado metabólico comprometido, como a uremia na DRC, pode resultar na modificação da expressão gênica mediada pela epigenética. Portanto, estabelece-se a chamada memória urêmica^60,61^. A metilação do DNA é o principal mecanismo pelo qual ocorre a progressão da DRC, com a regulação de genes críticos envolvidos na inflamação, fibrose e distúrbios metabólicos. Wing et al.^62^ estudaram pacientes afro-americanos com DRC e observaram metilação diferencial em genes como o RPTOR, um componente essencial da via de sinalização mTOR. O estudo comprovou que a hipermetilação do RPTOR levou à redução de sua expressão, o que inibiu a função do mTOR, essencial para o crescimento celular, a autofagia e a manutenção do equilíbrio energético.

Chu et al.^63^ realizaram ainda um estudo de associação em todo o epigenoma (EWAS) e constataram a hipermetilação do gene Klotho, um fator renoprotetor bem conhecido. A regulação negativa do Klotho, resultante do silenciamento dos genes, foi associada à deterioração prematura da função renal e ao aumento de estresse oxidativo, calcificação vascular e fibrose, levando, em última instância, ao agravamento dos desfechos da DRC. Além disso, Ko et al.^64^ investigaram o perfil de metilação em todo o genoma e detectaram uma extensa hipometilação global do DNA em pacientes com DRC, especialmente em genes relacionados à inflamação, como os genes da via NF-κB. Essa hipometilação epigenética promoveu a regulação positiva de citocinas pró-inflamatórias, provocando ainda mais disfunção imunológica e inflamação crônica na DRC. Em conjunto, esses dados indicam que as modificações epigenéticas atuam não apenas como biomarcadores de progressão da doença, mas também como alvos terapêuticos.

## Aplicações Clínicas e Direções Futuras

O futuro do manejo da DRC reside na identificação e no uso de novos biomarcadores, além dos tradicionais creatinina sérica e TFGe. Novos biomarcadores, como NGAL e KIM-1 urinários, têm demonstrado potencial na identificação de injúria renal antes do comprometimento funcional evidente. Em estudos clínicos, com desempenho bem superior ao basal, demonstrou-se que níveis urinários de NGAL >150 ng/mL indicavam lesão e transição de IRA para DRC com alta sensibilidade, enquanto o KIM-1 tem se mostrado consistente, a uma especificidade de 95%, em coortes de nefropatia diabética^65,66^. O receptor do ativador de plasminogênio tipo uroquinase solúvel (suPAR) também foi identificado como um biomarcador de inflamação sistêmica e progressão da DRC que oferece *insights* sobre os processos da doença além da lesão renal específica. Em análises multivariadas ajustadas, níveis elevados de suPAR (>3.040 pg/mL) previram de forma independente uma perda mais rápida da TFGe (quartil superior vs. inferior: -4,2 vs. -0,9 mL/min/1,73 m^2^ por ano). As taxas de DRC em cinco anos ocorreram em 41% dos pacientes com suPAR elevado e em 12% entre os grupos com suPAR baixo^67^. As tecnologias ômicas, especialmente proteômica, metabolômica e epigenômica, continuam a revelar assinaturas moleculares cada vez mais sofisticadas associadas ao risco de DRC.

Padrões de metilação do DNA, microRNAs circulantes e perfis de metabólitos constituem alguns candidatos promissores e não invasivos que surgiram para a estratificação de risco, distinguindo entre pacientes de alto risco e baixo risco para a progressão da DRC. A tradução desses biomarcadores em prática clínica, especialmente com o auxílio da inteligência artificial e algoritmos de *machine learning*, permitiria uma determinação de risco altamente individualizada, com potencial para intervenções mais precoces por parte dos nefrologistas e adequação da terapia com base nas características específicas da doença de cada paciente.

Além disso, abordagens terapêuticas baseadas em biomarcadores, nas quais o tratamento se baseia em perfis moleculares específicos, provavelmente darão início a uma nova era da medicina personalizada na nefrologia. Ao promover a transição de um manejo reativo para um proativo, os biomarcadores emergentes podem transformar o manejo da DRC, aprimorar a detecção precoce, retardar a progressão da doença e, em última instância, reduzir o ônus da incidência de DRT.

## Conclusão

Novos biomarcadores para a estratificação do risco de DRC têm o potencial de revolucionar o cuidado ao paciente, ao possibilitar o diagnóstico precoce, um prognóstico mais preciso e abordagens de tratamento personalizadas. Embora promissores, são necessários estudos de validação em larga escala para estabelecer seu uso clínico de rotina. A incorporação multidisciplinar desses biomarcadores nas estruturas clínicasexistentes permitiria explorar todo o potencial no manejo da DRC e os benefícios para os pacientes.

## Disponibilidade de Dados

O compartilhamento de dados não é aplicável, já que todas as informações sintetizadas nesta revisão estão disponíveis online.
